# The effect of early life events on glucose levels in first-episode psychosis

**DOI:** 10.3389/fendo.2022.983792

**Published:** 2022-12-05

**Authors:** Clemente Garcia-Rizo, Bibiana Cabrera, Miquel Bioque, Gisela Mezquida, Antonio Lobo, Ana Gonzalez-Pinto, Covadonga M. Diaz-Caneja, Iluminada Corripio, Eduard Vieta, Inmaculada Baeza, Maria Paz Garcia-Portilla, Miguel Gutierrez-Fraile, Roberto Rodriguez-Jimenez, Marina Garriga, Emilio Fernandez-Egea, Miguel Bernardo, PEPs GROUP

**Affiliations:** ^1^ Barcelona Clínic Schizophrenia Unit, Institute of Neuroscience, Hospital Clínic of Barcelona, University of Barcelona, Barcelona, Spain; ^2^ Centro de Investigación Biomédica en Red de Salud Mental (CIBERSAM), Madrid, Spain; ^3^ Institut d’investigacions Biomèdiques August Pi i Sunyer (IDIBAPS), Barcelona, Spain; ^4^ Department of Medicine and Psychiatry, University of Zaragoza, Instituto de Investigación Sanitaria Aragón (IIS Aragón), Zaragoza, Spain; ^5^ Hospital Universitario de Alava, Servicio de Psiquiatría, BIOARABA, University of the Basque Country, Vitoria, Spain; ^6^ Department of Child and Adolescent Psychiatry, Institute of Psychiatry and Mental Health, Hospital General Universitario Gregorio Marañón, IiSGM, School of Medicine, Universidad Complutense, Madrid, Spain; ^7^ Department of Psychiatry, Hospital de la Santa Creu i Sant Pau, Barcelona, Spain; ^8^ Bipolar and Depressive Disorders Unit, Hospital Clinic, Institute of Neuroscience, University of Barcelona, Barcelona, Spain; ^9^ Child and Adolescent Psychiatry and Psychology Department, Hospital Clinic of Barcelona, Institute of Neurosciences, University of Barcelona, Barcelona, Spain; ^10^ Department of Psychiatry, University of Oviedo, Oviedo, Spain. Instituto de Investigación Sanitaria del Principado de Asturias (ISPA), Oviedo, Spain; ^11^ Department of Psychiatry, Araba University Hospital, University of the Basque Country (UPV/EHU), Vitoria, Spain; ^12^ Neurosciences Department, Araba University Hospital, University of the Basque Country (UPV/EHU), Vitoria, Spain; ^13^ Instituto de Investigacion Sanitaria, Hospital 12 de Octubre (imas12), Madrid, Spain; ^14^ Department of Psychiatry, University of Cambridge, Addenbrooke’s Hospital, Cambridge, United Kingdom; ^15^ Cambridgeshire and Peterborough NHS Foundation Trust, Huntingdon, United Kingdom

**Keywords:** First-episode psychosis, glucose values, birth weight, fetal programming, thrifty psychiatric phenotype

## Abstract

First episode of psychosis (FEP) patients display a wide variety of metabolic disturbances at onset, which might underlie these patients’ increased morbidity and early mortality. Glycemic abnormalities have been previously related to pharmacological agents; however, recent research highlights the impact of early life events. Birth weight (BW), an indirect marker of the fetal environment, has been related to glucose abnormalities in the general population over time. We aim to evaluate if BW correlates with glucose values in a sample of FEP patients treated with different antipsychotics. Two hundred and thirty-six patients were included and evaluated for clinical and metabolic variables at baseline and at 2, 6, 12, and 24 months of follow-up. Pearson correlations and linear mixed model analysis were conducted to analyze the data. Antipsychotic treatment was grouped due to its metabolic risk profile. In our sample of FEP patients, BW was negatively correlated with glucose values at 24 months of follow-up [r=-0.167, p=0.037]. BW showed a trend towards significance in the association with glucose values over the 24-month period (F=3.22; p=0.073) despite other confounders such as age, time, sex, body mass index, antipsychotic type, and chlorpromazine dosage. This finding suggests that BW is involved in the evolution of glucose values over time in a cohort of patients with an FEP, independently of the type of pharmacological agent used in treatment. Our results highlight the importance of early life events in the later metabolic outcome of patients.

## Introduction

First episode of psychosis (FEP) is characterized by a wide variety of clinical symptomatology characterized by the presence of psychosis (delusions and hallucinations), with a pooled median point and 12-month prevalence of approximately four persons per 1,000 (1). Psychosis is a clinical construct in which, due to severe impairment of thoughts and emotions, a person is unable to distinguish between the internal experience of the mind and external reality. The British physician Thomas Willis initially described the association between mental health and abnormal glycemic homeostasis some centuries ago, stating that “diabetes was caused by sadness or long sorrow and other depressions”. Later, Henry Maudsley, a British psychiatrist described in his book *The Pathology of Mind*, that “Diabetes is a disease which often shows itself in families in which insanity prevails” (2). Many other authors delved into the association between psychosis and type 2 diabetes mellitus (T2DM) before the introduction of the first antipsychotic (3), chlorpromazine in 1952, after which the topic was relegated to the background. Nevertheless, the reduced life expectancy described in mental health disorders in recent decades has re-emphasized the historical connection.

FEP patients are diagnosed based on clinical symptomatology; in addition, they may be susceptible to increased morbidity (4, 5) and early mortality, mainly due to cardiovascular diseases and type 2 diabetes mellitus (T2DM) (6). Although the effect of antipsychotic treatments in the later development of T2DM has been widely reported in patients (7), glucose abnormalities and insulin resistance have been repeatedly described in patients before the use of any antipsychotic agent (8, 9). Notwithstanding this important research, the literature has not been able to confirm a specific genetic link between psychosis and T2DM despite independent findings (10). An environmental approach has tried to decipher this complex association, relying on the role of early life events in the later metabolic outcomes of patients, mostly with regard to T2DM (11, 12). The developmental origins of health and disease concepts (13), initially described by Professor David Barker (14), reflect that different early life events (famine, infections, stress during gestation) promote long-lasting changes in the metabolism of the fetus (through epigenetic changes in insulin and leptin homeostasis). These changes optimize the adaptation of the fetus to the maternal environment in the short term. However, if these circumstances modify over time (the stressful environment normalizes), previous metabolic changes can result in maladaptive behavior, and consequently the patient may develop metabolic abnormalities such as T2DM, which over time can lead to early mortality.

The previously described association between T2DM and psychosis suggests an abnormal glucose pathway at the onset of psychosis (15, 16). Several meta-analyses describe an increased prevalence of early life events in psychosis (17, 18); therefore from a theoretical perspective some patients might be at risk of developing T2DM, which would definitely increase after the use of antipsychotic agents. Indeed, several recent studies have proved a metabolic correlation between early life events and medical outcomes in psychosis, using a surrogate marker of the intrauterine environment, such as birth weight (BW). This approach was additionally proved in the general population, where BW correlated with later medical events in adulthood (14, 19). In a population-based cohort study, BW modified the effect of the correlation between BW and T2DM in patients with serious mental illnesses, and low and high BW were correlated in a quadratic form with higher prevalence of T2DM in patients diagnosed with schizophrenia (20). In another population-based cohort study including patients with schizophrenia among other diagnoses, BW was a risk factor for T2DM but did not modify the association with psychotropic medication (21). In a cohort of minimally treated patients with FEP, BW correlated and was associated with antipsychotic-induced weight gain over a two-year follow-up period (22); the same finding was found in another cohort of drug-naïve FEP patients treated with olanzapine over 18 weeks (23). In a transversal study assessing treatment-resistant patients diagnosed with schizophrenia, BW was associated with abdominal obesity (24). In a retrospective study, BW was associated with the onset of T2DM in a cohort of treatment-resistant schizophrenia patients (25). This could be referred to as the “metabolic imprinting” of early life events in psychosis, suggesting that both mental diagnosis and metabolic disturbances might behave as epiphenomena of early life events (12).

With the previous rationale, we aim to evaluate if BW correlates and is associated with glucose values in a cohort of FEP patients during a 24-month follow-up while taking into consideration antipsychotic treatment and other confounding factors.

## Material and methods

### Subjects

Sixteen Spanish centers with experience in performing and assessing diagnoses, using semi-structured interviews and clinical scales, and treating patients in the early phases of psychotic disorders, participated in the PEPs Project (26). The inclusion criteria for patients were age between 7 and 35 years, presence of a FEP in the last 12 months, and speak Spanish correctly. During the recruitment period (from April 2009 to April 2012), every patient who met the inclusion criteria and attended the inpatient or outpatient facilities was invited to participate in the study (27). The study was approved by the Board of Research and Ethics Committee of all participant centers and was performed in accordance with the ethical standards laid down in the 1964 Declaration of Helsinki and its later amendments. The rationale and the complete clinical protocol used in the PEP project has been previously published (28).

### Study assessments and biochemical determinations

At baseline, a complete medical history was taken. Birth weight (BW) was collected from maternal interviews. Body weight, height, and waist circumference were assessed at baseline and at 2-, 6-, 12-, and 24-month visits. Body mass index (BMI) is defined as body mass divided by the square of the body height and is universally expressed in units of kg/m^2^. Venous blood samples were collected by nursing staff in the morning (between 8:00 and 10:00) after fasting overnight and stored initially at 4°C. The antipsychotic mean daily dose was calculated in chlorpromazine equivalents (29).

We categorized the patients taking antipsychotic medication into three groups according to their antipsychotic-induced weight gain (AIWG) risk [high (olanzapine and clozapine), medium (quetiapine, risperidone, paliperidone), and low (aripiprazole, amisulpride, ziprasidone, haloperidol)] (30). As previously described, patients changed antipsychotic medication over the study period (31), and therefore we categorized with preference to higher risk: they were assigned to the high-risk group if they had ever been treated at any time with olanzapine or clozapine, and to the medium-risk group if they had ever been treated with risperidone, paliperidone, or quetiapine, but never with olanzapine or clozapine).

### Statistical analysis

The demographic and baseline clinical variables were described for the total sample and stratified by sex. For continuous variables, the mean and standard deviation was described. Categorical variables were analyzed by analysis of frequencies.

We used the Pearson correlation to evaluate the association between two continuous variables at every time point (as independent measures). Subsequently, and based on glucose findings at 24 months, we decided to use linear mixed model analysis (LMMA) to evaluate the glucose evolution over time (baseline, 2 months, 6 months, 12 months, and 24 months) while including the effect of time and other potential confounders, as we accept that individual data might not be independent at each time point.

LMMA is strongly recommended and a standard way to handle the dependencies of longitudinal data at hand; the model with maximum likelihood estimation has the advantage of coping with participant drop-out in an efficient way (32). LMMA also has the advantage of allowing the investigation of variability between patients (heterogeneity) and simultaneously adjusting for within-subject correlation. For repeated covariance type, the diagonal covariance matrix was selected.

The LMMA model used the glucose value over time as the dependent variable, while time, sex, antipsychotic drug type, BMI over the time period, chlorpromazine equivalents over the period time, age, and BW were used as independent variables.

To evaluate the level of the association between glucose values over the study period and BW, we used the predictive values derived from the LMMA. These were plotted to correlate with BW in order to describe graphically the predicted value of glucose values over time for given any BW.

Data were managed and analyzed with the IBM SPSS Statistics v.24.

## Results

Clinical and sociodemographic baseline characteristics of the sample are described in **Table 1**.

**Table 1:** Sociodemographic and clinical characteristics of the sample.

	Total sample	Female 35% (N=82)	Male 65% (N=154)
Age (years old)	23.51 ± (5.8)	25.03 ± (5.9)	22.70 ± (5.6)
Birth Weight (Kg)	3.28 ± (0.5)	3.16 ± (0.6)	3.34 ± (0.5)
Glucose value (mg/dL)	84.99 ± (18.0)	83.70 ± (17.0)	85.72 ± (18.6)
Body Mass Index (Kg/m^2^)	23.70 ± (4.6)	23.44 ± (5.7)	23.83 ± (3.9)
Chlorpromazine equivalent mean dose (mg/day)	553.71 ± (420.9)	446.67 ± (330.5)	610.12 ± (452.4)
Antipsychotic metabolic risk profile	High 39%/Medium 42% /Low 10% / No Antipsychotic 9%	High 34%/Medium 40% /Low 12% / No Antipsychotic 13%	High 41%/Medium 43%/Low 9% / No Antipsychotic 7%

For quantitative variables Mean ± (Standard Deviation).

For qualitative variables Percentages (frequencies).

Two hundred and thirty-six patients were included in the analysis at baseline. Pearson correlations between BW and glucose at baseline were [r=0.065, p=0.335, N=221], at 2 months [r=-0.042, p=0.567, N=187], at 6 months [r=-0.047, p=0.527, N=181], at 12 months [r=0.018, p=0.816, N=168], and at 24 months [r=-0.167, p=0.037, N=157].

Two hundred and twenty-six patients were included in the LMMA. Fixed effects are described for sex (F=3.47; p=0.063), age (F=8.31; p=0.004), time (F=0.34; p=0.848), antipsychotic drug type (F1.55; p=0.200), equivalent doses of chlorpromazine (F=1.04; p=0.308), BMI (F=25.66; p<0.001), and BW (F=3.22; p=0.073).

**Figure 1:**
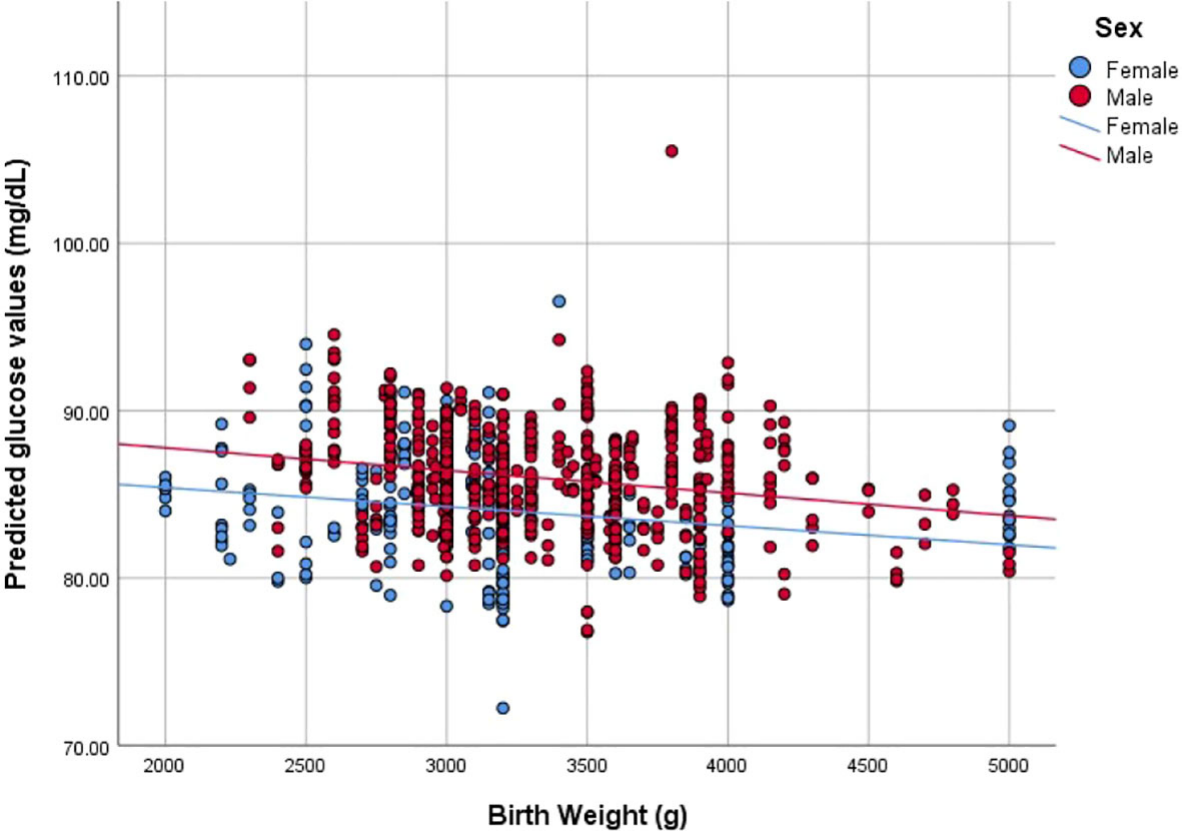
Correlation between Birth weight (g) and predicted glucose values (mg/dL) stratified by sex.

Predicted values were plotted to evaluate the association between BW and glucose values over the 24-month period. The model showed a similar significant pattern with linear (R^2 =^ 0.029, p<0.001) and quadratic correlation (R^2 =^ 0.029, p<0.001). When stratified by sex, correlations were as follows: for female participants, linear (R^2 =^ 0.045, p<0.001) and quadratic correlation (R^2 =^ 0.065, p<0.001), and for male participants, linear (R^2 =^ 0.047, p<0.001) and quadratic correlation (R^2 =^ 0.048, p<0.001) (**Figure 1**).

## Discussion

Our results confirm that BW correlates and shows a trend towards significance with glucose values at a 24-month period of follow-up in a cohort of FEP patients minimally treated before inclusion. The association holds despite diverse confounding factors such as type or dose of antipsychotic, BMI, sex, or age.

These findings suggest that early life events (represented by an indirect marker of the intrauterine milieu such as BW) play an important role in the metabolic abnormalities described in FEP, which could underlie the increased burden of morbidity and reduced life expectancy described at onset (6, 33).

Sex showed a trend towards significance, presenting higher values for male participants than female. Interestingly, male fetuses tend to grow faster than female at early stages making them more vulnerable to stressors (34) such as maternal stress (35). Age and BMI were also significantly associated with glucose values over time.

Several studies have described factors affecting antipsychotic-induced weight gain in FEP patients, which can be theoretically translated as factors of glucose abnormalities (4, 36). However, only a few studies have focused on the effect of early life events, either perinatal (20, 22–25) or postnatal (37, 38).

Our results reflect a negative relationship, with lower BW correlating with higher glucose values, which is similar to findings in a previous study where lower BW correlated with higher adiposity in treatment-resistant patients (24). Obstetric complications during gestation are associated with lower BW (39), while higher BW is associated with gestational diabetes (GDM). Methylation in several genes involved in metabolism, such as leptin or glucagon-like peptide receptors (40), or insulin-growing factors (41) have been described as potential pathways to explain the effect of low BW on later metabolic outcomes. Indeed, not only low BW but also GDM promote similar offspring methylation patterns, suggesting common genes and pathways linked to T2DM (42). As expected, abnormalities during gestation will promote two main effects: on one side, metabolic changes intra uterus will promote a risk of morbidity and early mortality (over time and in combination with resource-plentiful environment, antipsychotic side-effects, and unhealthy lifestyle), and on the other side, they will interfere in the normal neuronal maturation and migration, increasing the risk of developing psychosis (12). Indeed, previous obstetric complications have been described as affecting not only metabolism but also brain structure (43), cognition (44), and psychopathology (45).

Surprisingly, no differences in the pharmacological treatment arose between the groups evaluated based on their metabolic profile (30). This negative finding could be due to previous antipsychotic treatment, not only before entering the study but also in the initial 12 months, as the first year is a critical period for the development of cardiovascular risk factors (46). Indeed, in a sample of drug-naïve patients, differences in glucose values at the one-year follow-up appointment were not statistically significant in patients with different metabolic profiles who were taking one of three different antipsychotic medications (47).

There are several limitations associated with our study. Firstly, BW is a surrogate marker of the intrauterine environment and is affected by birth length and gestational age, which were not recorded and may have biased our results. Although several meta-analyses confirm the association between BW and glucose values in the general population (19, 48), recent research has shown the significance of the association related with gestational age, suggesting T2DM as a consequence of prematurity (49). Secondly, BW was gathered from maternal interviews, and despite a possible recall bias, BW has been described as an accurate measurement parameter in psychosis studies (50). Information regarding physical activity, dietary intake, and parental history of T2DM was not available, as well as other important prenatal events such as occurrence of gestational diabetes and maternal obesity. We included the antipsychotic drug type because of the potential AIWG risk; however, patients were receiving different levels of medication. In addition, other pharmacological agents were not included in the analysis; however, in a previous analysis of the sample, glucose values were not modified by concomitant pharmacological agents (4).

Our findings show a correlation between BW and glucose values over a 24-month follow-up period and a trend towards significance in the association between BW and glucose values over this period. However, we must highlight the fact that the number of subjects included was low compared with studies in the general population, and patients were relatively young. In the meta-analysis by Knop (19), most of the studies included population samples, while others included either young subjects evaluated with more sensitive measures, such as a glucose-tolerance test (51), or much older adults (52). Our results show that in our small sample of young FEP patients (patients were on average 23 years old), low BW correlates with higher glucose values, suggesting that those patients could develop glucose abnormalities over time.

Our results highlight the clinical importance of the prenatal environment to the later morbidity and early mortality risks of FEP patients.

## PEPs group

Sílvia Amoretti, Centro de Investigación Biomédica en Red de Salud Mental (CIBERSAM), Madrid, Spain; Department of Psychiatry, Hospital Universitari Vall d'Hebron; Group of Psychiatry, Mental Health and Addictions, Psychiatric Genetics Unit, Vall d'Hebron Research Institute (VHIR), Barcelona, Spain; Biomedical Network Research Centre on Mental Health (CIBERSAM), Spain; Universitat Autònoma de Barcelona, Barcelona, Spain; Ana Meseguer, Barcelona Clínic Schizophrenia Unit, Institute of Neuroscience, Hospital Clínic of Barcelona, University of Barcelona, Barcelona, Spain; Centro de Investigación Biomédica en Red de Salud Mental (CIBERSAM), Madrid, Spain; Institut d’investigacions Biomèdiques August Pi i Sunyer (IDIBAPS), Barcelona, Spain; David Fraguas, Centro de Investigación Biomédica en Red de Salud Mental (CIBERSAM), Madrid, Spain; Department of Child and Adolescent Psychiatry, Institute of Psychiatry and Mental Health, Hospital General Universitario Gregorio Marañón, IiSGM, School of Medicine, Universidad Complutense, Madrid, Spain; Carmen Moreno, Centro de Investigación Biomédica en Red de Salud Mental (CIBERSAM), Madrid, Spain; Department of Child and Adolescent Psychiatry, Institute of Psychiatry and Mental Health, Hospital General Universitario Gregorio Marañón, IiSGM, School of Medicine, Universidad Complutense, Madrid, Spain; Anna Alonso, Centro de Investigación Biomédica en Red de Salud Mental (CIBERSAM), Madrid, Spain; Department of Psychiatry, Hospital de la Santa Creu i Sant Pau, Barcelona, Spain; Eva Mª Grasa, Centro de Investigación Biomédica en Red de Salud Mental (CIBERSAM), Madrid, Spain; Department of Psychiatry, Hospital de la Santa Creu i Sant Pau, Barcelona, Spain; Jessica Fernandez, Centro de Investigación Biomédica en Red de Salud Mental (CIBERSAM), Madrid, Spain; Hospital Universitario de Alava, Servicio de Psiquiatría, BIOARABA, University of the Basque Country, Vitoria, Spain; Sainza García, Centro de Investigación Biomédica en Red de Salud Mental (CIBERSAM), Madrid, Spain; Hospital Universitario de Alava, Servicio de Psiquiatría, BIOARABA, University of the Basque Country, Vitoria, Spain; Maria Fe Barcones, Department of Medicine and Psychiatry, University of Zaragoza, Instituto de Investigación Sanitaria Aragón (IIS Aragón), Zaragoza, Spain; Centro de Investigación Biomédica en Red de Salud Mental (CIBERSAM), Madrid, Spain; Concepción de la Cámara, Centro de Investigación Biomédica en Red de Salud Mental (CIBERSAM), Madrid, Spain; Department of Medicine and Psychiatry, University of Zaragoza, Instituto de Investigación Sanitaria Aragón (IIS Aragón), Zaragoza, Spain; Julio Sanjuan, Centro de Investigación Biomédica en Red de Salud Mental (CIBERSAM), Madrid, Spain; INCLIVA, Universidad de Valencia, Hospital Clínico Universitario de Valencia, Spain; Hospital Clínico Universitario de Valencia, Spain; Mª Dolores Moltó, Centro de Investigación Biomédica en Red de Salud Mental (CIBERSAM), Madrid, Spain; INCLIVA, Universidad de Valencia, Hospital Clínico Universitario de Valencia, Spain; Antonio Bulbena, Institut de Neuropsiquiatria i Addiccions, Hospital del Mar, Barcelona, Spain; IMIM (Hospital del Mar Medical Research Institute), Barcelona; Department of Psychiatry and Forensic Medicine, Autonomous University of Barcelona UAB, Bellaterra, Spain; Purificación Salgado, Institut de Neuropsiquiatria i Addiccions, Hospital del Mar, Barcelona, Spain; IMIM (Hospital del Mar Medical Research Institute), Barcelona; Department of Psychiatry and Forensic Medicine, Autonomous University of Barcelona UAB, Bellaterra, Spain; Laura Montejo, Centro de Investigación Biomédica en Red de Salud Mental (CIBERSAM), Madrid, Spain; Institut d’investigacions Biomèdiques August Pi i Sunyer (IDIBAPS), Barcelona, Spain; Bipolar and Depressive Disorders Unit, Hospital Clinic, Institute of Neuroscience, University of Barcelona, Institut d’investigacions Biomèdiques August Pi i Sunyer (IDIBAPS), Centro de Investigacion Biomedica en Red de Salud Mental (CIBERSAM), Barcelona, Spain; Justo Pinzón, Bipolar and Depressive Disorders Unit, Hospital Clinic, Institute of Neuroscience, University of Barcelona, Institut d’investigacions Biomèdiques August Pi i Sunyer (IDIBAPS), Centro de Investigacion Biomedica en Red de Salud Mental (CIBERSAM), Madrid, Spain; Institut d’investigacions Biomèdiques August Pi i Sunyer (IDIBAPS), Barcelona, Spain; Child and Adolescent Psychiatry and Psychology Department, Hospital Clinic of Barcelona, Institute of Neurosciences, University of Barcelona, Barcelona, Spain; Josefina Castro, Centro de Investigación Biomédica en Red de Salud Mental (CIBERSAM), Madrid, Spain; Institut d’investigacions Biomèdiques August Pi i Sunyer (IDIBAPS), Barcelona, Spain; Child and Adolescent Psychiatry and Psychology Department, Hospital Clinic of Barcelona, Institute of Neurosciences, University of Barcelona, Barcelona, Spain; José Manuel Menchón, Psychiatry Department, Bellvitge University Hospital-IDIBELL, L'Hospitalet de Llobregat, Spain; Fernando Contreras, Psychiatry Department, Bellvitge University Hospital-IDIBELL, L'Hospitalet de Llobregat, Spain; Leticia García-Alvarez, Centro de Investigación Biomédica en Red de Salud Mental (CIBERSAM), Madrid, Spain; Department of Psychiatry, University of Oviedo, Oviedo, Spain. Instituto de Investigación Sanitaria del Principado de Asturias (ISPA), Oviedo, Spain; Leticia Gonzalez-Blanco, Centro de Investigación Biomédica en Red de Salud Mental (CIBERSAM), Madrid, Spain; Department of Psychiatry, University of Oviedo, Oviedo, Spain. Instituto de Investigación Sanitaria del Principado de Asturias (ISPA), Oviedo, Spain; Rafael Segarra, Centro de Investigación Biomédica en Red de Salud Mental (CIBERSAM), Madrid, Spain. Department of Neurosciences, University of the Basque Country, Cruces University Hospital, Biocruces Bizkaia Health Research Institute, Vizcaya, Spain; Neurosciences Department, Araba University Hospital, University of the Basque Country (UPV/ EHU), Vitoria, Spain; Arantzazu Zabala, Centro de Investigación Biomédica en Red de Salud Mental (CIBERSAM), Madrid, Spain. Department of Neurosciences, University of the Basque Country, Cruces University Hospital, Biocruces Bizkaia Health Research Institute, Vizcaya, Spain; Isabel Morales-Muñoz, Instituto de Investigacion Sanitaria, Hospital 12 de Octubre (imas12), Madrid, Spain; Mónica Dompablo, Instituto de Investigacion Sanitaria, Hospital 12 de Octubre (imas12), Madrid, Spain; Judith Usall, Parc Sanitari Sant Joan de Déu, Institut de Recerca Sant Joan de Déu, Sant Boi de Llobregat, Barcelona, Spain Centro de Investigación Biomédica en Red de Salud Mental (CIBERSAM), Madrid, Spain; Anna Butjosa, Parc Sanitari Sant Joan de Déu, Institut de Recerca Sant Joan de Déu, Sant Boi de Llobregat, Barcelona, Spain Centro de Investigación Biomédica en Red de Salud Mental (CIBERSAM), Madrid, Spain; Fundació Sant Joan de Déu, Institut de Recerca Sant Joan de Déu, Esplugues de Llobregat, Barcelona, Spain; Salvador Sarró, Centro de Investigación Biomédica en Red de Salud Mental (CIBERSAM), Madrid, Spain; FIDMAG Germanes Hospitalàries Research Foundation, Barcelona, Spain; Ramón Landín-Romero, Neuroscience Research Australia, Sydney, NSW, Australia School of Medical Sciences, University of New South Wales, Sydney, NSW, Australia ARC Centre of Excellence in Cognition and its Disorders, Sydney, NSW, Australia; Ángela Ibañez, Centro de Investigación Biomédica en Red de Salud Mental (CIBERSAM), Madrid, Spain; Department of Psychiatry, Hospital Universitario Ramón y Cajal, IRYCIS, Universidad de Alcalá, Madrid, Spain; Manuel J Cuesta, Department of Psychiatry, Complejo Hospitalario de Navarra, Pamplona, Spain; IdiSNA, Navarra Institute for Health Research, Pamplona, Spain; Vicent Balanzá-Martínez Centro de Investigación Biomédica en Red de Salud Mental (CIBERSAM), Madrid, Spain; Teaching Unit of Psychiatry, Department of Medicine, University of Valencia, Valencia, Spain.

## Data availability statement

The raw data supporting the conclusions of this article will be made available by the authors, without undue reservation.

## Ethics statement

The PEPS study was conducted in accordance with the ethical principles stated on the Declaration of Helsinki. The protocol was reviewed and approved by ethics committees at each participating center. A written and signed informed consent was obtained from all participants or their legal guardians after providing a full explanation of the study's procedures.

## Author contributions

CG-R designed the current study, wrote the first draft of the manuscript, and with MG undertook the statistical analysis. BC, MBi, GM, MG and EF-E managed the literature searches. AL, AG-P, CD-C, IC, EV, IB, MG-P, MG-F, RR-J and MBe contributed to the earlier versions of the manuscript. All authors contributed to the article and approved the submitted version.

## Funding

This study is part of a coordinated-multicentre Project, funded by the Ministerio de Economía y Competitividad (PI08/0208; PI11/00325; PI14/00612), Instituto de Salud Carlos III, Fondo Europeo de Desarrollo Regional. Unión Europea. Una manera de hacer Europa, Centro de Investigación Biomédica en Red de salud Mental, CIBERSAM, by the CERCA Programme/Generalitat de Catalunya and Secretaria d’Universitats i Recerca del Departament d’Economia I Coneixement (2017SGR1355). This study has been funded by Instituto de Salud Carlos III (ISCIII) through the project “PI20/00661” and co-funded by the European Union. This work was developed (in part) at the Centro Esther Koplowitz (Barcelona). This project is also grateful for the support of the Institut de Neurociències, Universitat de Barcelona. This research was supported by CIBER -Consorcio Centro de Investigación Biomédica en Red- (código CIBER), Instituto de Salud Carlos III, Ministerio de Ciencia e Innovación and Unión Europea – European Regional Development Fund.

## Acknowledgments

We are extremely grateful to all subjects who took part in this study. The authors thank Ferran Torres (Platform for Medical Statistics, Institut d’Investigacions Biomèdiques August Pi i Sunyer (IDIBAPS), Hospital Clinic, Barcelona) and Cristina V. Oliveira (Barcelona Clinic Schizophrenia Unit, Hospital Clinic, Barcelona) for their methodological support. IB thanks the Instituto de Salud Carlos III-ISCIII for its support (INT19/00021). AG-P has received grants and served as consultant, advisor or CME speaker for the following entities: the Spanish Ministry of Science and Innovation CIBERSAM, the Ministry of Science Carlos III Institute, the Basque Government, and the Stanley Medical Research Institute. CD-C holds a Juan Rodés grant JR19/00024, Instituto de Salud Carlos III, Spanish Ministry of Science and Innovation, and has received honoraria from AbbVie, Sanofi, and Exeltis. EV has received grants and served as consultant, advisor or CME speaker for the following entities; the Brain and Behaviour Foundation, the Spanish Ministry of Science and Innovation CIBERSAM, the Seventh European Framework Programme ENBREC, and the Stanley Medical Research Institute. IB has received , research support from Fundacio´n Alicia Koplowitz and grants from the Spanish Ministry of Health, Instituto de Salud Carlos III. RR-J has been a consultant for, spoken in activities of, or received grants from: Instituto de Salud Carlos III, Fondo de Investigación Sanitaria FIS, Centro de Investigación Biomédica en Red de Salud Mental CIBERSAM, Madrid Regional Government S2010/BMD-2422 AGES. CG-R has obtained research funding from the Spanish Ministry of Science and Education, the Spanish Ministry of Economy and Competiveness, Centro de Investigación Biomédica en Red de Salud Mental CIBERSAM, by the Government of Catalonia, Secretaria d’Universitats i Recerca del Departament d’Economia i Coneixement 2014SGR441, Foundation European Group for Research In Schizophrenia EGRIS, and the 7th Framework Program of the European Union.

## Conflict of interest

CG-R has received honoraria/travel support from Abbott, Adamed, Angelini, Cassen-Recordati, Janssen-Cilag and Lundbeck. MBi has been a consultant for, received grant/research support and honoraria from, and been on the speakers/advisory board of has received honoraria from talks and/or consultancy of Adamed, Angelini, Ferrer, Janssen-Cilag, Lundbeck, Neuraxpharm, Otsuka, Pfizer and Sanofi. AL has received honorarium or travel support from Lundbeck and Sanofi. AG-P has received grants and served as consultant, advisor or CME speaker for the following entities: Almirall, AstraZeneca,Bristol-Myers Squibb, Cephalon, Eli Lilly, Glaxo-Smith-Kline, Janssen-Cilag, Ferrer, Johnson & Johnson, Lundbeck, Merck, Otsuka, Pfizer,Sanofi-Aventis, Servier, Shering-Plough, Solvay, and and Wyeth. CD-C has received honoraria from AbbVie, Sanofi, and Exeltis. IC has received research grants and served as consultant, advisor or speaker for the companies Otsuka and Ferrer. EV has received grants and served as consultant, advisor or CME speaker for the following entities: AB-Biotics, Allergan, Angelini, AstraZeneca, Bristol-Myers Squibb, Dainippon Sumitomo Pharma, Farmindustria, Ferrer, Forest Research Institute, Gedeon Richter, Glaxo-Smith-Kline, Janssen, Lundbeck, Otsuka, Pfizer, Roche, Sanofi-Aventis, Servier, Shire, Sunovion, and Takeda. IB has received honoraria or travel support from Otsuka, and Angelini and Janssen. MG-P has been a consultant to and/or has received honoraria/grants from Angelini, Alianza Otsuka- Lundbeck, Instituto de Salud Carlos III, Janssen-Cilag, Lundbeck, Otsuka, Pfizer, and SAGE Therapeutics. MG-F has been on the speakers/advisory board of Janssen-Cilag. RR-J has been a consultant for, spoken in activities of, or received grants from: Janssen Cilag, Lundbeck, Otsuka, Pfizer, Ferrer, Juste. MB has been a consultant for, received grant/research support and honoraria from, and been on the speakers/advisory board of AB-Biotics, Adamed, Angelini, Casen Recordati, Janssen-Cilag, Menarini, Roviand Takeda.

The remaining authors declare that the research was conducted in the absence of any commercial or financial relationships that could be constructed as a potential conflict of interest.

## Publisher’s note

All claims expressed in this article are solely those of the authors and do not necessarily represent those of their affiliated organizations, or those of the publisher, the editors and the reviewers. Any product that may be evaluated in this article, or claim that may be made by its manufacturer, is not guaranteed or endorsed by the publisher.
